# Applying Gamification Principles and Therapeutic Movement Sequences to Design an Interactive Physical Activity Game: Development Study

**DOI:** 10.2196/38133

**Published:** 2022-12-16

**Authors:** Hyungsook Kim, David Michael O'Sullivan, Seong Hee Chung

**Affiliations:** 1 Department of Cognitive Sciences Hanyang University Fusion Technology Center Seoul Republic of Korea; 2 Graduate School of Public Policy Hanyang University Seoul Republic of Korea; 3 Hanyang Digital Healthcare Center Hanyang University Seoul Republic of Korea; 4 Division of Sport Science Pusan National University Pusan Republic of Korea

**Keywords:** home workout, mobile assistant, movement, physical activity, depression

## Abstract

**Background:**

Depression is a severe illness that has accelerated with the spread of COVID-19 and associated lockdowns. As a result, reported physical activity has substantially decreased, further increasing depressive symptoms.

**Objective:**

This study aims to explain the use of gamification principles to develop content for an interactive physical activity game for depression based on clinically proven depression diagnostic criteria.

**Methods:**

We discuss related work in this field, the game design framework, the users’ depression severity, how we customize the contents accordingly, the gradual progression of the game to match exercise principles, and user flow optimization.

**Results:**

We provide a brief description of each of the games developed, including instructions on how to play and design aspects for flow, audio, and visual feedback methods. Exergames (interactive physical activity–based games) stimulate certain physical fitness factors such as improving reaction time, endurance, cardiovascular fitness, and flexibility. In addition, the game difficulty progresses based on various factors, such as the user’s performance for successful completion, reaction time, movement speed, and stimulated larger joint range of motions. Cognitive aspects are included, as the user has to memorize particular movement sequences.

**Conclusions:**

Mental health issues are linked to behavior and movement; therefore, future physical activity–based interactive games may provide excellent stimulation for inducing user flow, while physical activity can help train various physical fitness factors linked to depression.

## Introduction

The adverse effects of the COVID-19 pandemic have substantially affected the physical and mental health of the public worldwide [1,2]. Lockdowns and restrictions have resulted in an increase in the lack of physical activity and depression [3,4]. Health care providers are responsible for addressing these issues. Until recently, exercise was a prominent nonpharmaceutical therapeutic method to supplement pharmacology and additional invasive treatments for mental health issues. Exercise has been shown to be effective in ameliorating mental health–related issues such as depression [5,6] and dementia [7,8]. Despite the significant effectiveness of exercise as a complementary method to improve the symptoms of mental health issues, it is challenging for people to remain engaged in and motivated to continue exercising [9,10]. Therefore, it is vital to provide users with physical activity–based immersive and fun interventions to optimize retention. Furthermore, a vast amount of research indicates that even minimal increases in physical activity lead to highly significant improvements in the health status of the general population [11,12]. Research shows a dose-response relationship between an increase in physical activity and health-related benefits.

Gamification is a well-known process that increases fun, motivation, engagement, and flow during an activity. Its principles have been used in various areas to stimulate more immersive interactions during activities. Gamification is a proven method that can help increase physical activity, with many mobile apps and video game–based physical activity intervention products having their effectiveness proven through many studies [13-16]. Recent studies classify the types of gamification [17] into competition and cooperation, self-goal, storytelling type, and positive/negative reinforcement [18]. Competition, which provides incentives to increase physical activity, was reported to be the most effective method, and the gamification factor of support or collaboration did not significantly increase physical activity [16]. An additional essential element of gamification is aesthetics, with realistic graphic visual and sound effects that provide more fun to users by creating aesthetically pleasing environments with sensory stimulation. Reinforcement and rewards are reported to increase the level of immersion of game users, increase voluntary participation motivation, and increase the frequency and time of intervention participation [19-22]. In video game interventional therapy, high interactivity and better visual and auditory stimulation have been reported to ameliorate therapeutic effects [23]. In addition, among depression-related software in which gamification is applied, more than 16 apps have demonstrated direct therapeutic effects on depression in clinical research [24].

To develop our game, the movement sequence was designed to include various movement patterns for each body part, especially the arms, legs, and torso. The contents we developed provide a structure to stimulate the progression from basic motion to movement, that is, a sequence of motions. When the user performs (experiences) the game, a simple motion is repeated that strengthens the muscles surrounding the involved limbs. Tai-Chi–related research [25] shows that repetitive lifting of bodyweight and isometric movements (maintaining a static pose) can strengthen muscles and improve cardiovascular endurance. In addition, as exergames use bodyweight only, the level of movement intensity is similar to that of Tai-chi (where a practitioner moves through a series of poses). Therefore, we expect a similar physiological response in muscle strength and cardiovascular enhancement with only nonintense movements in the upper and lower limbs [24,26].

Furthermore, we expect that repeated movement patterns, especially in an open and upright posture in the vertical plane, can help improve the user’s mood [27,28], reduce fatigue, and help improve focus for people with mild-to-moderate depression [29]. Movements with large ranges of each joint (ie, open or space-consuming posture) are defined as high power or powerful poses. This expansive, open pose (also called “power posing”) is a method of nonverbal communication that improves not only the psychological state but also the neurohormones [30-32]. A user’s positive experience reinforces the fun of participating and retains positive feedback in their memory. We also expect the positive effects of the regular use of this exergame to help increase users’ daily physical activity levels due to improved physical function, cardiovascular fitness, and muscle strength.

Exergames, which are the innovation of using technology to stimulate exercise, show that gamification can help participants engage and enjoy exercise more. In particular, exergames are found to be effective for youth with obesity than for the older population with dementia [33]. In a systematic review, exergames have been shown to reduce depression symptoms, but their effectiveness depends on depression severity, the number of sessions, and game type (high and low playfulness) [24]. Although exergames have been shown to help increase the fun of exercise, to our knowledge, no exergames have been developed for the treatment and management of depression. Therefore, in this study, the objective was to explain how we used gamification principles to develop the contents of an exergame (interactive physical activity game) controlled by Azure Kinect (Microsoft Corporation) for depression based on clinically proven depression diagnostic criteria.

## Methods

### The Physical Activity Game Design Framework

**Figure 1:**
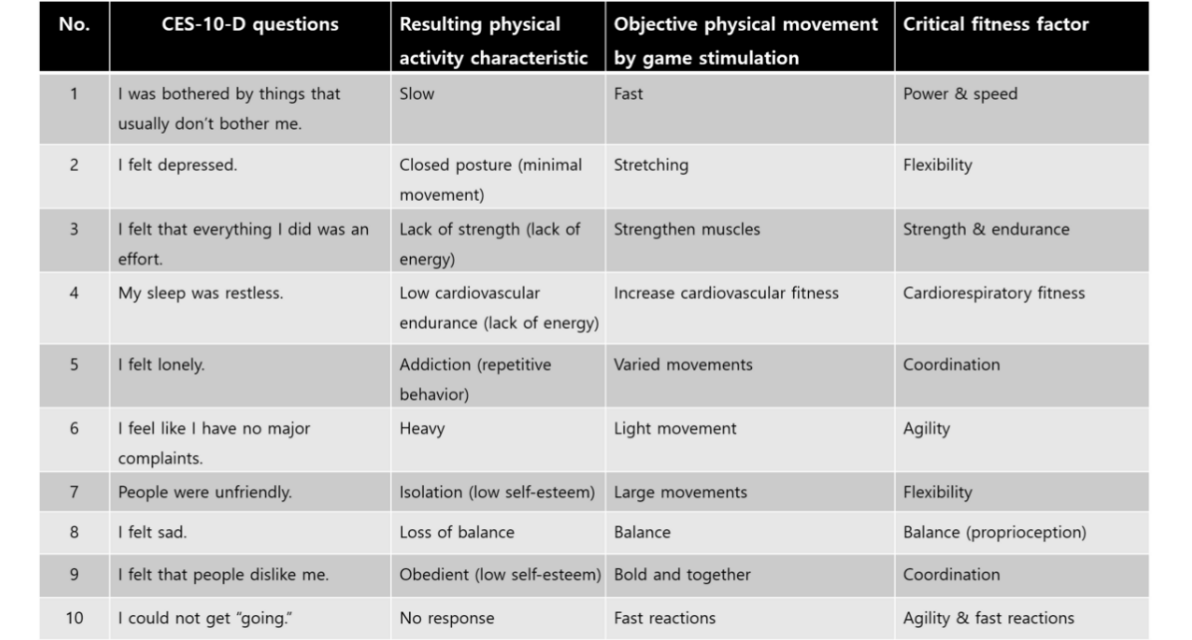
Extraction of the 6 core fitness factors to stimulate the resulting physical activity characteristic of the CES-10-D questionnaire. CES-D-10: 10-item Center for the Epidemiological Studies of Depression Short Form.

A total of 6 diagnostic criteria (Diagnostic and Statistical Manual of Mental Disorders, Fifth Edition [DSM-5]) and 6 core fitness factors (10-item Center for the Epidemiological Studies of Depression Short Form [CES-D-10]) were selected (details in Figure 1) through a review of the DSM-5 depression diagnostic criteria and the CES-D-10. We developed exergames with 6 different physical activities based on these 6 core fitness factors (coordination, cardiovascular endurance, balance, reaction time, muscular endurance, and agility).

**Figure 2:**
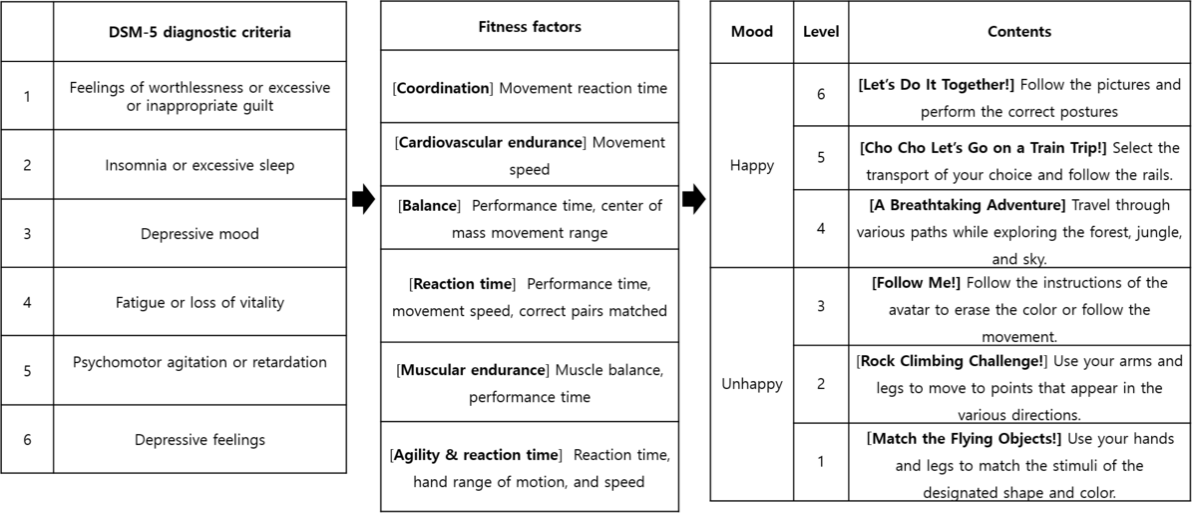
Extraction of the fitness factors from the DSM-5 criteria and CESD-10-D to develop 6 specific physical activity game tasks. CES-D-10: 10-item Center for the Epidemiological Studies of Depression Short Form; DSM-5: Diagnostic and Statistical Manual of Mental Disorders, Fifth Edition.

The general procedure for mapping the 6 core factors when developing a game for each of them is as follows: First, the behavioral characteristics of the daily life of patients with depression were derived for each core factor. The specific behaviors of people with depression (depressive trait behaviors) were derived from these core factors and summarized through a literature review and detailed consultations with neurologists and mental health specialists. The core factors were then linked to fitness factors through a series of consultations with psychiatric clinicians and physical activity experts (Figure 2). Second, specific movement patterns that could strengthen each fitness factor linked to depressive behaviors were designed.

In this section, the theory and principles for developing the exergames are explained. The first step in game development was to extract the related fitness factors from the DSM-5 diagnostic criteria and the CES-10-D scale for depression. Then, specific exergames were developed based on the fitness factors needed to be trained. For example, the participants were required to follow the correct postures with their upper and lower bodies for coordination training, as demonstrated in the game.

### Extraction of Physical Activity Patterns From the CES-10-D

Items 1 and 2 are associated with unhappiness and depressive moods, which are reported to produce the symptom of slow movements, that is, psychomotor retardation (longer reaction times) with slow brain potentials [34]. For example, various studies have shown that gait patterns associated with sadness and depression are characterized by slower walking speed, reduced arms sign, and reduced vertical head movements [35,36]. In addition, people with depression and sadness showed larger lateral swaying and had a more slumped posture [35]. A recent review about gait, balance tasks, and posture in major mental illnesses such as depression and anxiety also indicated that evaluation and treatment programs should include specific physical movement characteristics, such as balance, gait pattern, and posture [37].

Feeling depressed is also associated with poor posture, that is, sitting in a slouched position [29]. A novel study [29] investigated whether changing posture could reduce negative affect and fatigue in people with mild-to-moderate depression undergoing a stressful task. The study found that adopting an upright posture appeared to increase positive affect, reduce fatigue, and decrease self-focus in people with mild-to-moderate depression. Besides, participants in the upright group spoke significantly more words than the usual posture group, used fewer first-person singular personal pronouns, but more sadness words. Comparable effects of posture were also shown to affect the ability of college students to significantly recall negative memories easily when they were sitting in a slouched posture than when they were sitting in an erect posture [38]. The authors recommended that therapists should teach their clients posture awareness and that they should sit up in an erect position during their daily lives to increase positive affect and decrease depressive symptoms [38]. Michalak et al [39] reported that even relatively minor changes in the motoric system (posture) can affect the cognitive biases in depression and suggested training patients in mindful body awareness to foster the understanding of the interplay between bodily and emotional processes.

Items 3 and 4 are questions related to fatigue, with depression strongly linked to a lack of physical activity, especially in late-life depression [40]. Mänty et al [41] showed that there are significant reductions in the isometric muscle strength (ie, knee extension, body extension, and handgrip) of older people, which highly correlate with gait speed. Similarly, Szlejf et al [42] reported that depression is associated with sarcopenia due to the low muscle strength in middle-aged and older adults. In addition, a study by Song et al [43] indicated that exercise programs such as Tai-Chi can have positive effects on middle-aged and older females by increasing their physical function in terms of muscle strength, flexibility, and endurance, as well as their depression scores. The importance of physical exercise in treating depression and the strong correlation between increased muscle strength and reduced feelings of depression have already been highlighted [44].

Item 5 is related to feelings of loneliness, with physical activity reported to contribute to a decrease in the feeling of loneliness [45]. Moreover, in a population-based observational cohort study that used comparable annual data collected between 2015 and 2019 to evaluate the effect of COVID-19 on depression and its contributing factors, the decrease in physical activity was identified as a risk factor for the worsening mental health. The authors also recommended policies to target potentially modifiable risk factors such as participating in physical activity [46]. As loneliness may be a reason why people do not want to participate in physical activity, it is important to have an easily accessible method for people to become more physically active in their daily lives [47].

Agitation is an easily observable phenomenon associated with depression [48] and can be characterized using the observation scale developed by Hurley et al [49], which includes total body movements, up and down movements, repetitive motion in place, outward motions, high pitched or loud words, repetitive vocalization, and negative words. Repetitive behaviors are shown to be related to anxiety and depression in youth with autism spectrum disorder [50].

Item 6 is a heavy feeling and so we recommend light agile movements to improve this mood. Michalak et al [35] reported on the negative effect of sadness and depression on movement/gait, describing the gait of walkers with depression as heavy, because they have larger lateral sway and walk in a slumped posture with more pronounced slower, lower arm swing and more slumped posture. We propose that light and agile movements should be introduced to counteract the feeling of heaviness. The feeling of heaviness is reported widely throughout the literature to be associated with depression, suggesting that biological and neurological mechanisms can affect physical activity, which can be explained by neurophenomenological studies and enactive dimensions of embodiment [51]. These movement-based embodied contemplative practices have been used to help alleviate depressive symptoms, with interventions using mindfulness mediation, yoga, Tai-Chi, dance, and movement therapy [51]. Recently, in a meta-analysis investigating the effectiveness of dance movement therapy as an intervention/therapy for depression, moderate effect sizes were found, and in all the cases the depression scores decreased, with the decrease continuing for several months even after the completion of the therapy [51]. Furthermore, in a multicenter, randomized control trial in Finland, dance movement therapy—performed 2 times per week for a total of 10 weeks—with standard care was shown to be an effective treatment for depression, with medium effect sizes reported at the 3-month follow-up time [52].

Item 7 focuses on reducing feelings of isolation by encouraging large movements to help improve the feeling of social belonging. Decreases in daily physical activity and increased sedentary time are related to an increased risk of ill health; furthermore, poor well-being is associated with isolation among older men and women [53]. In this study we propose that providing an opportunity to engage in physical activity and encouraging large movements could help relieve the feelings of isolation. In addition, the context and social benefits of engaging in regular leisure-time activities and physical activity are deemed to explain the reduction of depression symptoms [54]. A recent study of 3052 US adults revealed that people who could not meet the recommended daily physical activity levels as a result of the pandemic reported worse depression, loneliness, stress, and less positive mental health overall [55].

For item 8, as poor balance is associated with depressive symptoms, we focused on developing user’s balance and proprioceptive neuromuscular system. Qi and colleagues [56] showed that muscular paralysis and balance problems were significantly associated with increased severities of depression (odds ratio 13.5 and 2.9, respectively), and recommended that testing for changes in balance and muscular paralysis could be useful biomarkers for assessing depression severity that could be administered regularly for monitoring patients with major depressive disorders [56]. Similarly, the “gait and brain study” showed that depressive symptoms may amplify balance problems in older adults with mild cognitive impairment during sensorimotor challenges [57]. In addition, the study showed increases in postural sway during open and closed eye conditions for patients with more depressive symptoms [57]. Likewise, Casteran et al [58] associated balance with depression by recording more sway among both male and female elderly participants with depression. As significant depressive symptoms may affect balance in the older population, it may thus also increase the potential for the risk of falls, which can be fatal and have a substantial impact on people with and without depression, making it more difficult to be physically active and independent.

As item 9 focuses on the person’s self-esteem behavior, we hypothesized that trying to coordinate movements with an avatar may help the participant feel closer to people. The relationship between exercise, self-esteem, and depression is highlighted in numerous publications [59,60]. In children with obesity, exercise alone as a treatment was shown to be effective in reducing the feeling of low self-esteem and improving depressive symptoms [60]. In addition, the role of self-esteem in depression is reported to be a predicting factor; in other words, patients with higher gains in self-esteem over the treatment period of cognitive behavioral group therapy had strong linear declines in depressive symptoms [60]. Exercise was also shown to be effective in the older population to increase self-esteem, reduce depressive symptoms, and improve overall quality of life [61]. Many studies [51,52] included dance therapy with slow cyclical movements as an intervention, and concluded that the soothing effect of dance therapy can have positive effects on mood. Despite the vast number of studies highlighting the positive effects of physical activities/exercise on self-esteem and depressive symptoms, the exact mechanism is not well discussed [62,63]. Future studies should therefore focus on the effects that specific types of physical activity have on the various physiological mechanisms that increase self-esteem, and therefore, reduce depressive symptoms.

Item 10 focuses on the amotivation of people to exercise. For this scenario, we suggest an intervention that encourages, almost forces, a participant to move as a natural reaction to a certain stimulus. Therefore, the contents of this intervention should trigger a rapid reaction movement that should be gamified to help the participant enter flow and forget their amotivation to do any physical activity. Studies have suggested that just inducing someone to initiate a movement could help them perform more physical activity [64]. As a result of the physical activity performed, the reward system (Behavioral Activation System theory) is activated and the participant feels more at ease and experiences an improved mood [65], as it significantly reduces anger, confusion, fatigue, tension, and vigor. In addition, participation in an exercise program reduces negative mood. Research shows that slow rhythmical movements at the frequency of the respiratory sinus arrhythmia (approximately 0.1 Hz) will help the participant feel “being in the groove” and is associated with more efficient blood circulation and increases in heart rate. Furthermore, these movements are shown to have a positive effect on the automatic nervous system [66]. Although there is not much research on the effect of explosive fast reaction time movements, we hypothesize that because it is the opposite to what the participant is used to, it should help initiate movement and eventually improve mood.

### Assessment of Depressive Severity to Customize User’s Level of Difficulty

The user’s game level was customized according to the 9-item Patient Health Questionnaire (PHQ-9) score, as shown in Table 1 and Figure 2. The user with the most severe depression symptoms, with a PHQ-9 score between 22 and 27, had to perform sets 1-1, 1-2, 1-3 then 2-3, up to 6-3, for a total of 8 sets. If users had a PHQ-9 score between 10 and 15, they had to perform sets 3-1, 3-2, 3-3 up to 6-3, for a total of 6 sets. If users had a PHQ-9 score between 1 and 3, they only had to perform sets 6-1 (warm-up), 6-2 (slowly increased intensity), and 6-3 (main exercise intensity), for a total of 3 sets. Based on the gamification principles, each set lasted approximately 5 minutes, which was shown to be the optimal time for someone to maintain concentration during a game [67].

**Table 1:** Allocation of the games required according to the severity of diagnostic score.

Mood and level		PHQ-9^a^ score		CES-10-D^b^ score
**Happy**				
	6	1-3	0	
	5	4-6	1-2	
	4	7-9	3-4	
**Unhappy**				
	3	10-15	5-6	
	2	16-21	7-8	
	1	22-27	9-10	

^a^PHQ-9: 9-item Patient Health Questionnaire.

^b^CES-D-10: 10-item Center for the Epidemiological Studies of Depression Short Form.

### Customized Exergames Matrix

**Figure 3:**
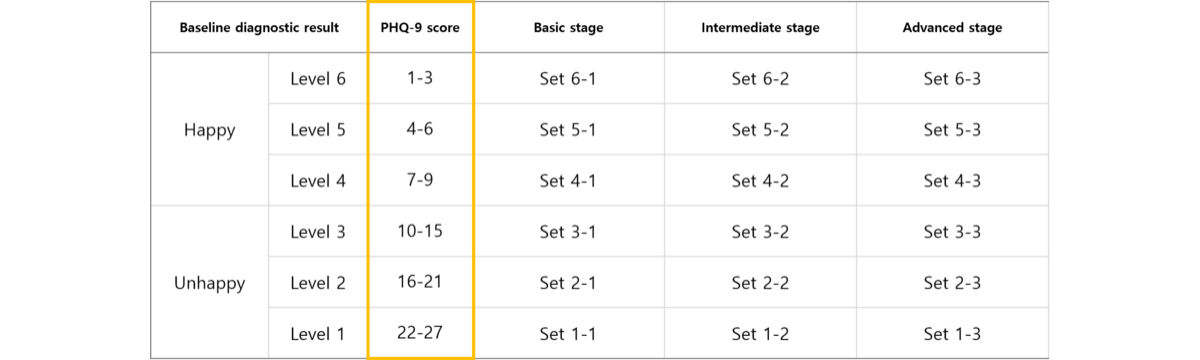
Sequence of games according to the PHQ-9 score. PHQ-9: 9-item Patient Health Questionnaire.

Based on the principles of physical activity training methods, the interactive game has basic, intermediate, and advanced stages (Figure 3). The basic stage allowed the participants to warm-up and stretch. The intermediate stage increased the movement range and speed of the required moves for the participant to progress gradually. The advanced stage was performed only after the basic and intermediate stages had been completed fully, and it required a full range of upper and lower body movements with high intensity and relative speed.

### Gamification for Player’s Flow

Gamification has been used to motivate users to practice games daily. The primary motivation for designing these games was to have fun and invoke a flow experience. They were designed to stimulate the users to continue, and it was crucial that the levels were neither too hard nor too easy; therefore, we assigned difficulty to each level according to the user’s ability and previous performance. Each of the presented games has pass and fail criteria to provide users with a sense of achievement to help motivate them, which will enable them to perform to their fullest potential so that they progress. In addition, after each attempt at a stage, the user is provided with game-dependent feedback: score, reaction times, movement speeds, and the number of correct postures. The game visuals and audio stimulation were designed to be aesthetically pleasing and easy to understand to help maintain immersion and make the game more user-friendly.

## Results

### Game 1: Collect the Space Treasure

In this game, the user must collect all the space treasure that is flying toward them using their hands and legs. Based on the advice of an expert group comprising game designers, mental health practitioners, and user interface/design experts, various shapes and colors were selected with the main aim to help increase interest in the game. As the level increases, the number and types of shapes and the speed at which they approach increase. Furthermore, the user’s range of movement is progressively increased from the objects being closer to the body to within the reach of the body without moving the trunk and finally to moving both arms and trunk to reach the objects. Each level takes up to 5 minutes to complete, with meteorites, stars, and flying saucers appearing sequentially in the basic level. At the intermediate level, there is a mixed appearance of meteorites, stars, and flying saucers, and at the advanced level, there is a mixed appearance of meteorites, stars, and flying saucers and colors.

**Figure 4:**
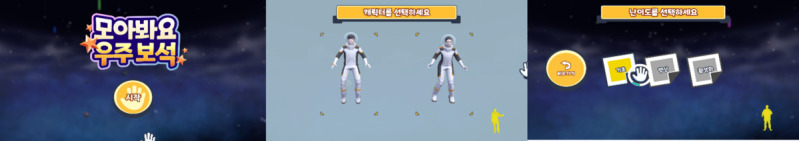
Game’s opening scene (left), user selecting their avatar (center), and user selecting their level (right).

At the start of the game, the user selects the space that suits their avatar’s attire and then their level (Figure 4). There are 149 target objects at the basic level, from which 70 must be matched correctly for successful completion. There are 220 target objects at the intermediate level, from which 110 must be matched correctly for successful completion. There are 315 target objects at the advanced level, from which 150 must be matched correctly for successful completion.

**Figure 5:**
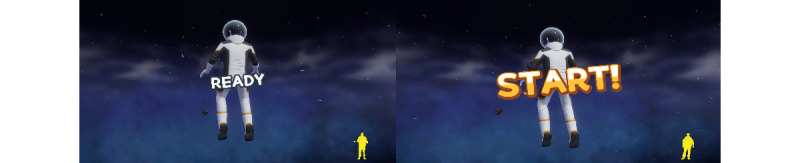
Guidance to get ready (center) and begin (right) with sound effects and music in the background to help maintain immersion.

Next, the user reads the game instructions on how to play and is guided though the process of getting ready and starting (Figure 5). Currently, the instructions are in the Korean language, as the test bed is located in Korea.

**Figure 6:**
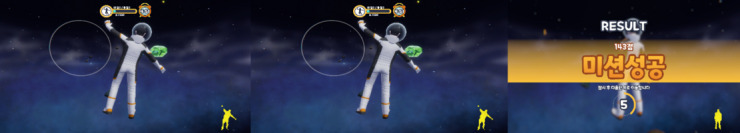
Reaching to the right (left), double-handed movement expanding to the full range of movement (center), and mission success screen (right).

The game then begins with an alien saying, “Let’s go to match some flying objects.” After matching the objects, as illustrated in Figure 6, the result is displayed as a mission success or failure. Here, one can see a mixture of combinations with the increasing range of movement and complexity by using both hands and following a sequence of movements (Figure 6). The user’s reaction time should improve as they have to match the objects in specific sequences and train their cardiovascular system owing to constant movement.

### Game 2: Rock Climbing Challenge

**Figure 7:**
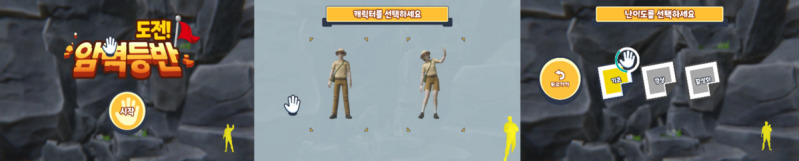
Game’s opening scene (left), user selecting their avatar (center), and user selecting their level (right).

**Figure 8:**
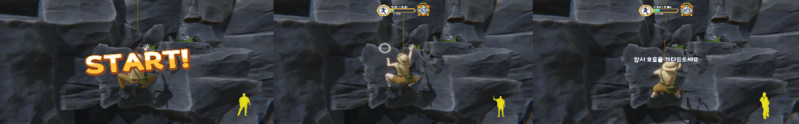
Starting screen to prepare user for the game (left), with guidance indicated with a circle (center). After a few minutes of exercise there is a time to recover and the user is encouraged to stretch and take a breath (right).

In this game, the user must maintain rock-climbing–type poses to advance up the wall (Figures 7 and 8). Muscle endurance is required in this game as the user must maintain certain poses, such as balancing on 1 leg and reaching. Initially, the poses use only the left and right hands; however, as the levels increase, the arms and legs must move together in more challenging poses. The complexity of the poses and the required range of movement increase as the game progresses. This game should help users improve their strength by requiring them to hold poses for a few moments. In addition, their joint range of motion will be increased when performing elongated stretching movements.

The user is required to select their avatar and the level of difficulty (Figure 7). After reading the instructions on how to play, the game begins (Figure 8). Figure 8 illustrates the sequence of movement required by the player to proceed to the next required pose. The user must move the hands and feet in the correct direction and pose for the level to proceed, and there will be movement noise to indicate that the sequence is correct and a visual stating “Good.”

There are 3 levels, each of which takes approximately 5 minutes to complete. There are 50 poses at the basic level, and each must be held for 4 seconds; 25 poses must match correctly for successful completion. There are 60 poses at the intermediate level, and each must be held for 3 seconds; 30 poses must match correctly for successful completion. There are 75 poses at the advanced level, and each must be held for 2 seconds; 40 poses must match correctly for successful completion.

### Game 3: Follow Me

**Figure 9:**
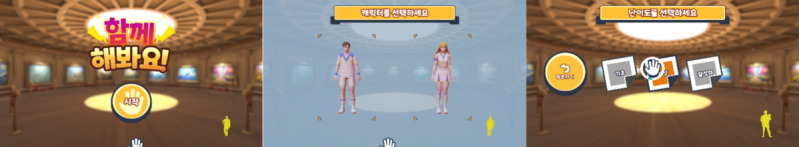
Game’s opening scene welcoming you to the art museum (left), selection of avatar (center), and selection of levels, from basic to advanced (right).

**Figure 10:**
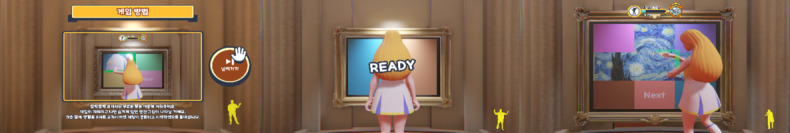
Game’s how to play screen (left), ready to play screen (center), and basic level (right).

**Figure 11:**
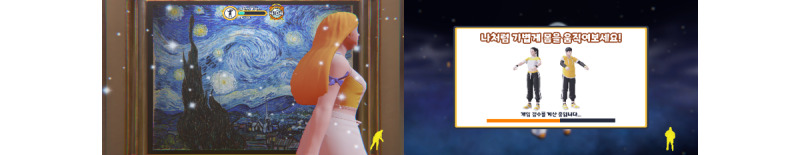
Indication that first painting is finished (left); user is instructed to rest and stretch for a few minutes (right).

In this game, the user must follow the avatar’s movement by erasing the coverings on top of the painting to reveal the original painting underneath (Figures 9-11). As with the previous games, the user begins by selecting the avatar (male or female) from the 3 levels (basic, intermediate, and advanced). The game begins after the instructions are displayed. The painting is divided into different blocks, allowing the user to erase the coverings to reveal the original painting underneath. As the level progresses, more squares (3×3 to 5×5) covering the painting appear, which requires more movement from the user to erase the painting coverings. The primary difference between the basic, intermediate, and advanced levels is that the user at the basic level only erases the coverings of the underlying art in their preferred order. However, for intermediate and advanced levels, the user must erase the coverings in separate movements, which are a combination of erasing a few coverings. In addition, the movements required to erase the covering takes more movement, and the user needs to move progressively further away from their standing position and reach up and down to various heights. Through repeated use of this game, the user’s range of motion should increase due to reaching up, down, left, and right in multiple directions (Table 2).

**Table 2:** Progression of levels and difficulty in game 3.

Levels	Number of physical activity movements	Pass criteria
Basic	4 seconds for 2 coverings (1×2) changes to 3 seconds for 4 spaces (2×2) to be erased 4 seconds for 9 coverings (3×3) performed 2 times 2 seconds for 16 coverings (4×4) performed 2 times changes to 2 seconds for 20 coverings (4×5) performed 2 times 16 seconds (randomly divided) performed 4 times	60/121 need to be correct
Intermediate	12 seconds per movement (a combination of coverings; 4-4-4); a total of 12 movements 6 seconds per movement (2-2-2); a total of 6 movements 12 seconds per movement (4-4-4); a total of 5 movements 6 seconds per movement (2-2-2); a total of 10 movements	15/33 need to be correct
Advanced	12 seconds per movement (4-4-4); a total of 4 movements 6 seconds for each movement (2-2-2); a total 32 movements 4 seconds per movement (simultaneous); a total of 15 movements	25/51 need to be correct

### Game 4: A Breathtaking Adventure

**Figure 12:**
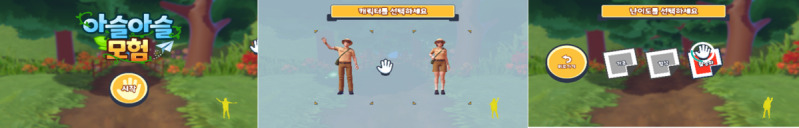
Game’s opening scene welcoming the user (left), selection of avatar (center), and selection of levels, from basic to advanced (right).

**Figure 13:**
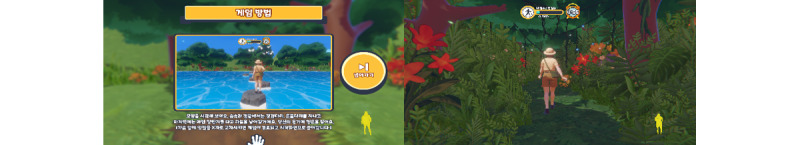
Game’s how to play screen (left). The basic level showing walking on a path (right).

**Figure 14:**
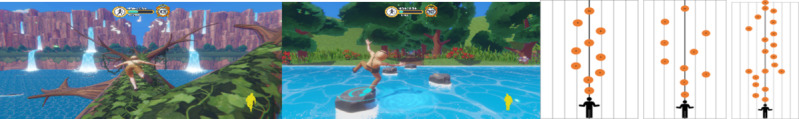
Game progresses to walking over narrow bridges (left), advanced-level walking across stepping stones (center), advanced-level walking on different paths, including narrow, wide, and more complex (right).

In this game, the user must navigate through the allocated paths, moving within a beautiful natural background (Figure 12). As with the previous games, the user begins by selecting the avatar (male or female) from the 3 levels (basic, intermediate, and advanced). The game begins after the instructions are displayed (Figure 13). The user progresses through the levels by first walking along an easy path, which then slowly becomes more challenging as they are required to pass narrow bridges, swaying rope bridges, and even crossing stepping stones (Figure 14). The user’s balance is improved by playing this game, as they must control their steps and directions to navigate. In addition, the user must avoid various obstacles and must move left or right to do so. The user must have 80/160, 40/80, and 40/85 successful steps at the basic, intermediate, and advanced levels, respectively. There is background noise such as natural sounds, special visual effects, and audio and visual guidance to make the game more intuitive and user-friendly and help immerse in and enjoy it.

### Game 5: Cho Cho Let’s Go on a Trip!

**Figure 15:**
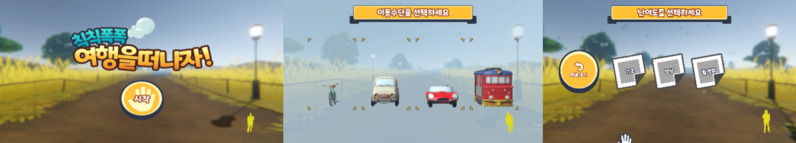
Game’s opening scene welcoming you to go on a trip (left), selection of mode of transport (center), and selection of levels, from basic to advanced or exit (right).

**Figure 16:**
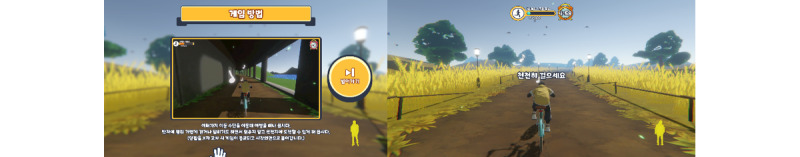
Game’s how to play screen (left) and basic-level cycling on a path (right).

**Figure 17:**
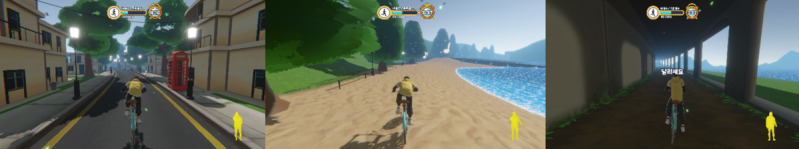
Game progresses to traveling through towns and cities (left), traveling by the sea (center), and traveling through the countryside (right).

In this game, the user must walk or jog on the spot to control the speed and movement of a train (Figures 15-17). The game strengthens the cardiovascular system because the user must keep walking or jogging slowly on the spot. Users can select from various modes of transport such as bicycles, cars, or trains. The levels progress by increasing the users’ movement speed as they move through the numerous natural backgrounds, such as by the sea, through the countryside, or city. The basic level starts with slow walking, normal speed walking, and then a slow jog, requiring the user to maintain continuous movements for more than 120 seconds out of 250 seconds to proceed to the next level. At the intermediate level, it progresses with slow walking, normal speed walking, a slow jog, and then a normal speed jog, with the user required to maintain movement for 180 continuous seconds out of 240 seconds. For the advanced level, the user performs interval-style training with a slow walk (30 seconds), light run (30 seconds), fast walk (30 seconds), run (30 seconds), slow walk (30 seconds), fast walk (30 seconds), light jog (30 seconds), and finishing with a run (60 seconds). The movement speeds were controlled by the tempo of the audio: 70 bpm for a slow walk (Adagio), 118 bpm for a fast walk (Allegretto), 156 bpm for a light run (Vivace), and 168 bpm for a run (Presto). There was 5-10-second rest between each of the required poses. The criteria for passing the most advanced stage 3 required the user to maintain movement for at least 200 seconds out of the 270 seconds.

### Game 6: Let’s Do It Together

**Figure 18:**
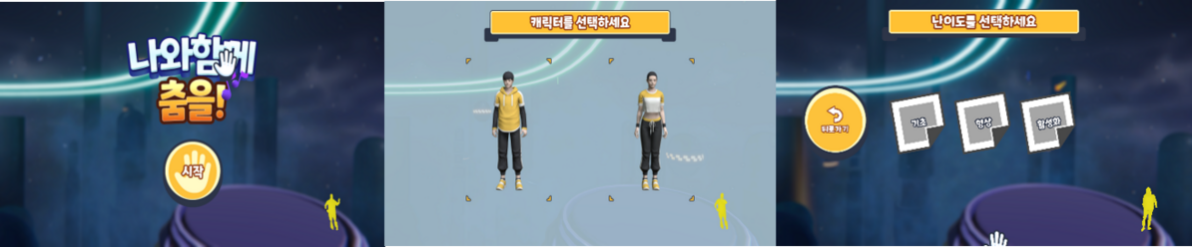
Game’s opening scene welcoming you to dance together (left), selection of avatar (center), and selection of levels, from basic to advanced or exit (right).

**Figure 19:**
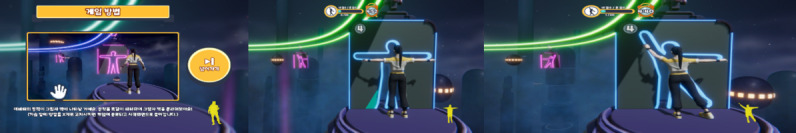
Game’s how to play screen (left), ready to play by matching the shape coming toward the player (center), and another and more difficult shape to match (right).

**Figure 20:**
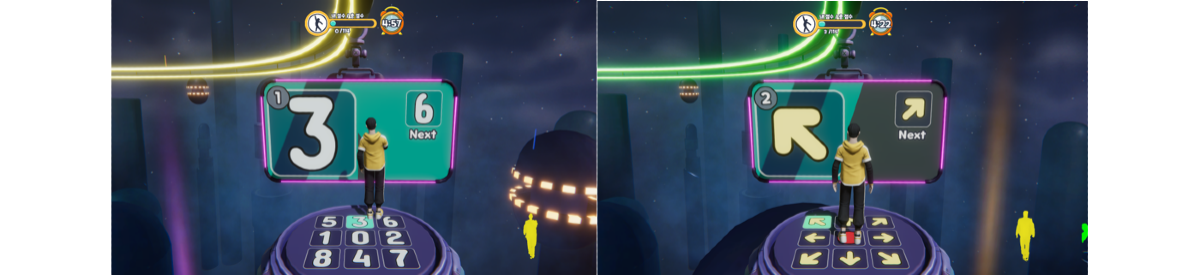
Visual feedback for movement on the ground numbers and arrows (left to right).

**Figure 21:**
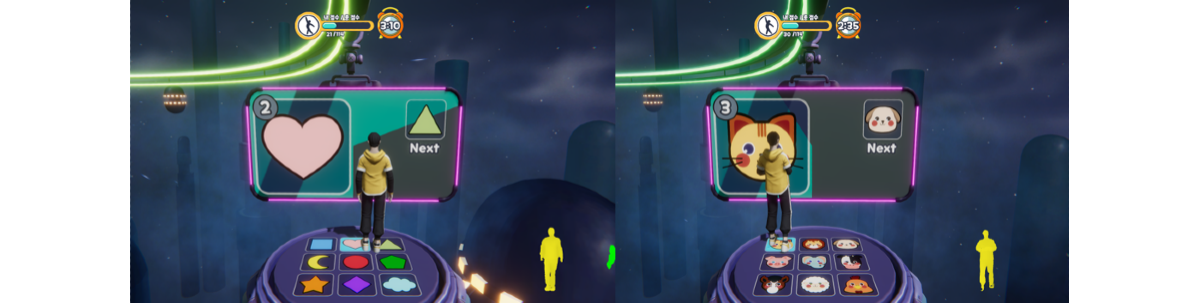
Visual feedback for movement on the ground shapes and animals (left to right).

In this game, the user must maintain postures so that their avatar can pass through the advancing walls with particular body shapes (Figures 18 and 19). The game starts with the user selecting their avatar (male or female) and then selecting levels 1, 2, or 3 (Figure 18). Instructions on how to play appear once the user understands and selects the next button to start the game. At the basic and intermediate levels, the user must maintain the particular poses to pass through the advancing walls, as shown in Figure 19. At the advanced level, the user must move according to the instructions on the screen. The movement at this level is not in particular poses; however, they must move on the ground left, right, forward, back, and in a combination of numbers, arrows, shapes, and animals (Figures 20 and 21). In the game, 11 motions appear randomly and are repeated. A movement is recognized as a success when it maintains a synchronization rate of 80%, according to the level. For the basic stage, the success criteria were 15 correct movements out of 30. For the intermediate stage, the success criteria were 22 correct movements out of 43, and for the advanced stage, the success criteria were to match 57 out of 114 pictures in total.

## Discussion

### Principal Findings

This article provides a detailed framework for applying the gamification principles to develop the contents of an exergame for depression based on clinically proven depression diagnostic criteria. The diagnostic criteria we selected to provide a structure for developing a systematic approach for treating depression were based on the DSM-5 depression diagnostic criteria and CES-D-10. Based on these criteria, we propose diverse physical activity–based interventions focusing on various related fitness factors, such as cardiovascular factors and strength.

We are currently seeking Institute Review Board approval to help determine the feasibility of the content and users’ experience. In this planned pilot test, we will obtain feedback on the usage of each of the games and their associated contents and visual and audio feedback, which will provide qualitative feedback for the service user to help improve the content through a refinement of the intervention. Upon completion of a series of tests and retests with relevant stakeholders, a randomized controlled trial will be conducted to check the effectiveness of the treatment or intervention and its cost-effectiveness relative to the standard methods of treatment and intervention.

A major strength of digital solutions is the exponential expandability of technology to reach users; however, this comes with the need for users to have digital literacy to use the content and initial investment in the hardware. A major limitation is the users’ need for hardware that is compatible with the developed content. Currently, having access to depth cameras is challenging; however, we are confident that, as they are instrumental and there is a growing amount of evidence demonstrating their vast applications in the field, they will continue to be developed and made more accessible in the future.

This study is beneficial as it provides a structured framework for developing digital content based on physical activity interventions for solving various mental health issues. By leveraging digital solutions, we hope that the overall cost of treatment for mental health issues will be reduced and that the suggested solution will be expandable for an exploding population that requires the management of mental health, particularly in the post-COVID-19 pandemic era.

### Conclusions

This study highlights the application of gamification principles to develop interactive physical activity content for a depression intervention game. In addition, we provide a structured methodology for developing content associated with depression movement characteristics (kinematics). Further research is required to determine the effectiveness and user experience of an exergame to prevent, manage, and treat depression in a range of low- to high-severity participants.

## Abbreviations

CES-D-10: 10-item Center for the Epidemiological Studies of Depression Short Form
DSM-5: Diagnostic and Statistical Manual of Mental Disorders, Fifth Edition
PHQ-9: 9-item Patient Health Questionnaire
